# ﻿A new Neotropical ant species of genus *Linepithema* Mayr (Hymenoptera, Formicidae, Dolichoderinae) with partial revision of the *L.fuscum* group based on males

**DOI:** 10.3897/zookeys.1160.95694

**Published:** 2023-05-09

**Authors:** Stefano Cantone, Andrea Di Giulio

**Affiliations:** 1 Department of Science, University ‘Roma Tre’, Viale G. Marconi, 446, 00146 Rome, Italy University ‘Roma Tre’ Roma Italy; 2 NBFC, National Biodiversity Future Center, Palermo 90133, Italy NBFC, National Biodiversity Future Center Palermo Italy

**Keywords:** External genitalia, *Linepithemapaulistana*, male ants, taxonomic status, winged ants

## Abstract

The genus *Linepithema* was erected by Mayr (1866) for his male-based species *L.fuscum*. In this study a new species is described also based on male morphology, *L.paulistana***sp. nov.**, collected in the city of São Paulo, Brazil, which is attributed to the *fuscum* group (Formicidae: Dolichoderinae). *Linepithemapaulistana***sp. nov.** is the only species of *fuscum* group present in the eastern part of South America. It is easily distinguishable from the other species of the group because of the presence of a triangular volsellar tooth, which is distally situated between the digitus and the basivolsellar process. By using SEM and optical microscopy, the external genitalia of *L.paulistana***sp. nov.** were analyzed and illustrated and some characters and previous interpretations have been re-evaluated in the *Linepithemafuscum* group. The male external genitalia are also comparatively analyzed in three species representative of the three *Linepithema* species groups, those of *fuscum*, *humile*, and *neotropicum*. The present work confirms that the morphological characters of male ants, especially those of male external genitalia, are effective for the identification of genera or species. Given the discrete morphological differences between the external genitalia of the *fuscum* group and the other species of this genus, a re-evaluation of the generic status of *Linepithema* is suggested.

## ﻿Introduction

The Neotropical ant genus *Linepithema* Mayr, 1866 (Formicidae: Dolichoderinae) includes 20 species that are widely distributed in Central and South America and the Caribbean (Wild 2007; Bolton 2022). Workers of this genus are monomorphic, and the males and queens are winged. The best known and most studied species is the invasive Argentine ant *L.humile* Mayr, 1868, which shows a cosmopolitan distribution due to its ability to adapt to different climatic conditions and to the plasticity of its reproductive strategies (Ward 1987; Aron et al. 1994, 2001; Aron 2001; Roura-Pascual et al. 2004; Menke and Holway 2006). The genus was erected based on males collected in Lima, Peru, which was described as *Linepithemafuscum* Mayr, 1866. Shattuck (1992a) subdivided the dolichoderine genus *Iridomyrmex* Mayr, 1862, transferring the Neotropical species to *Linepithema*, including *L.humile*, based on the examination of workers associated with males. In his generic review of Dolichoderinae, Shattuck (1992b) provided a diagnosis of *Linepithema* for the worker, queen, male, and the larval stage, and divided the genus into two male-based species groups, those of *fuscum* and *humile*.

*Linepithema* was later revised by Wild (2007), who followed Shattuck’s species-group classification and suggested that the *fuscum* group may be monophyletic, and the *humile* group may be paraphyletic, as it lacks distinct synapomorphies. Subsequently, Wild (2009), confirmed the monophyly of the *fuscum* group via molecular phylogenetics, and subdivided the *humile* group into three species groups: *humile* group, *iniquum* group, and *neotropicum* group. Unfortunately, *L.fuscum* could not be included in this phylogenetic analysis. According to Wild (2007, 2009), the *fuscum* group includes seven species, two of which, *L.cryptobioticum* Wild, 2007 and *L.flavescens* Wheeler & Mann, 1914, have unknown males. The highly distinctive characters of the males of the *Linepithemafuscum* group, compared to the other species groups, have been highlighted by both Shattuck (1992b) and Wild (2007), and mainly refer to: i) the submarginal cells of the forewings; ii) the ventral petiolar process; iii) the pygostyle; and iv) the paramere and volsella, which are parts of the external genitalia. Notably, the female castes of *L.fuscum* remain unknown, with the intersex association based solely on geography (Wild 2007), and no COI DNA barcode analysis was performed (Wild 2009).

In the present study we describe a new species of *Linepithema*, collected in the city of São Paulo (Brazil), based on males. Due to its genitalic form, we attribute the species to the *fuscum* group. The morphology of the new species is described by using optical and scanning electron (SEM) microscopy; these are the first SEM illustrations of a fuscum group male and include figures of the external genitalia. The male genitalia are compared to three species, representing three of the four species groups (*fuscum*, *humile*, and *neotropicum*). The morphological interpretation and terminology used for external genitalia in previous *Linepithema* descriptions is revised. Finally, an updated key to males of the species *Linepithema* in the *fuscum* group is presented, and a new species distribution map of the *Linepithemafuscum*-group is supplied.

## ﻿Materials and methods

### ﻿Material examined

Males of *Linepithemapaulistana* sp. nov., *L.humile*, and *L.neotropicum* Wild, 2007 were captured in São Paulo city (Brazil), 23°35'16"S, 46°38'55"W, altitude 800 m a.s.l. via two light traps (“Luiz de Queiroz” model), equipped with 15-watt UV black-blue lamps. The traps were left in the same position, attached to the same tree at 3 m and 7 m from the ground, kept active continuously from 01 August 2012 to 01 September 2014 and checked weekly (Cantone 2018b), with the specimens preserved in 70% ethanol. We identified the specimens using the works of Mayr (1866), Emery (1912), Shattuck (1992b), and Wild (2004, 2007). We had the following material at our disposal: *Linepithemapaulistana* sp. nov. (*fuscum* group), 12 male specimens collected from March to August 2013, and in January, February, and June 2014; *L.humile* (*humile* group), ten male specimens collected in April 2012, September to December 2013, and March 2014; and *L.neotropicum* (*neotropicum* group), 20 male specimens collected in the months of October to December 2012, January to April 2013 and July to December 2013. The holotype (MZUR3-HF0001) and seven paratypes (unique ID codes MZUR3-HF0002 to MZUR3-HF0008) of *L.paulistana* sp. nov., and specimens of *L.humile* and *L.neotropicum* are deposited in the Museum of Zoology of ‘Roma Tre’ University (Rome, Italy; **MZUR3**); two paratype males (MZUR3-HF0009 and MZUR3-HF0010) will be deposited in the Museum of Zoology of São Paulo University (**MZUSP**).

### ﻿Morphological analysis

The identifications and dissections were performed with Leica MZ12 (Leica Microsystems, Wetzlar, Germany) and Olympus SZX16 (Olympus, Tokyo, Japan) stereomicroscopes equipped respectively with Olympus Highlight 2100 and Olympus KL1500 LCD fiber optic lights. Dissected specimens were mounted on slides in Canada balsam and examined with an Olympus BX51 (Olympus, Tokyo, Japan) microscope. Optical micrographs of slide mounted specimens were taken with an Olympus BX51 microscope equipped with an om-d e-m5 digital camera (Olympus, Tokyo, Japan) with either a 10 × or 20 × objective. Remaining pictures of holotype were acquired with a Zeiss Axio Zoom V16 (Carl Zeiss AG; Oberkochen, Germany) and an Axiocam 503 (Carl Zeiss Microimaging Gmbh, Jena, Germany) equipped with Led dual spot-lights Photonic Optische (Vienna, Austria). Scanning electron microscopy was performed at L.I.M.E. lab (University of Roma Tre, Rome, Italy). Samples were dehydrated in a graded ethanol series (70%, 85%, 95%, 30 min each followed by 100% for 2 h), critical point dried (Balzer Union CPD 030 unit), mounted on aluminum stubs with a conductive adhesive carbon disk, sputtered with a thin layer (30 nm) of gold in a Emithech K550 sputter coater (Emithech, Kent, UK), and analyzed with a Zeiss Gemini 300 field emission SEM microscope at a voltage of 5 kV (Carl Zeiss AG, Jena, Germany).

The terminology used in this study represents a combination between classical studies on Hymenoptera and more specific studies on Formicidae and it based on Snodgrass (1941) for the external genitalia; Mason (1986) for the wing venation; Schulmeister (2001, 2003) for the external genitalia; Yoshimura and Fisher (2007, 2009, 2011) for the male general morphology and wing venation; Perfilieva (2010) for the wing venation; Boudinot (2013, 2015, 2018) for the external genitalia and mesosoma; Barden et al. (2017) for the external genitalia; Cantone (2017, 2018a) and Cantone and Von Zuben (2019) for the wing venation; Delsine et al. (2019) for the general morphology of Formicidae; Beutel et al. (2020) for the legs; Richter et al. (2019, 2020, 2021) for the head. When conflicting terminology was found, we prioritized the most recent reference studies applied to the family Formicidae.

### ﻿Measurements

In order to make comparisons with the other species of the genus *Linepithema*, measurements of *L.paulistana* sp. nov. follow the system of Wild (2007). We provided all measurements for the holotype and the morphometric variation (minimum and maximum) based on ten specimens (holotype and nine paratypes). In Table 1 we compared these measurements with those of all species of the *fuscum* group with known males.

**Table 1.** The most relevant morphological characters that differentiate the males of the *Linepithemafuscum* group from the males of the other *Linepithema* species. This table is based on the comparative tables by Shattuck (1992b) and Wild (2007). The new or re-evaluated diagnostic characters of the *fuscum* group resulting from this study are indicated in bold.

Morphological characters	Male *Linepithemafuscum* group	Male *Linepithema*, other species
**ventral petiolar process**	**slightly developed (Figs 1D, 3D)**	**well developed (Fig. 5A, B)**
forewings	two submarginal cells (Fig. 1E)	one submarginal cell
**proctiger**	**well developed (Fig. 4A**–**C)**	**slightly developed (Fig. 5C**–**D)**
pygostyles	very long (Fig. 4A)	short (Fig. 5C)
basimere	strongly thinned dorsally and shortened distally (Fig. 4C, F)	developed dorsally and distally (Fig. 5C, D)
telomere	narrow and very long ribbon-like, digitiform distally (Fig. 4A–C)	short and lobe form (Fig. 5C, D)
**basivolsellar process**	**reduced (Fig. 4C, D)**	**developed (Fig. 5E, F)**
digitus	dorsal and very long (Fig. 4B)	medial and short (Fig. 5C, D)
**cuspis**	**lateral and very reduced (Fig. 4D)**	**lateral and developed (Fig. 5C, D)**
**valviceps lamina**	**dentate ventral edge straight (Fig. 1J)**	**dentate ventral edge strongly convex and rounded (Fig. 5G, H)**

### ﻿Abbreviations for morphological characters

**HL** Head length, in full face view. The midline distance from the level of the maximum posterior projection of the margin of the head (not including the ocelli) to the level of the most anterior projection of the anterior clypeal margin.

**HW** Head width, in full face view, the maximum width of the head posterior to the compound eyes.

**MOD** Median ocellus diameter.

**SL** Antennal scape length, measured from the apex of the first antennal segment to the base, excluding the radicle.

**FL** Profemur length, in posterior view, measured along the longitudinal axis from the apex to the junction with the trochanter.

**LHF** Metafemur length.

**LHT** Metatibial length, in dorsal view, measured along the longitudinal axis from the apex to the level of the lateral condyles, excluding the medial proximal condyle.

**LHTa** Metabasitarsus length.

**EL** Eye length, in full face view, the length of the compound eye along the longitudinal axis.

**EW** Eye width, with eye held in focal plane facing the viewer, the maximum transverse width of the compound eye.

**MML** Maximum mesosomal length, the distance from the maximum anterior projection of the mesosoma to the maximum posterior projection of the propodeum.

**WL** Forewing length, the maximum distance between the insertion of the sclerotized wing veins to the distal margin of the wing.

**WHL** Hindwing length, the maximum distance between the insertion of the sclerotized wing veins to the distal margin of the wing

**ES** Eye size index. 100 × EL × EW

**CI** Cephalic index. 100 × HW/HL

**SI** Scape index. 100 × SL/HL

**OI** Ocular index. 100 × EL/HL

**WI** Wing index. 10 × WL/MML

**FI** Femoral index. 100 × FL/MML

## ﻿Results

### ﻿Class Insecta Linnaeus, 1758

**Order Hymenoptera Linnaeus, 1758**

**Family Formicidae Latreille, 1809**

#### ﻿Subfamily Dolichoderinae, Forel, 1878

##### Genus *Linepithema* Mayr, 1866

###### ﻿ Linepithema paulistana sp. nov.

Taxon classificationAnimaliaHymenopteraFormicidae

00EB98C0-4500-57D1-B1F2-192AA90411BF

https://zoobank.org/48DC08BF-0E4B-4946-9142-F26224830832

**Figure 1.**
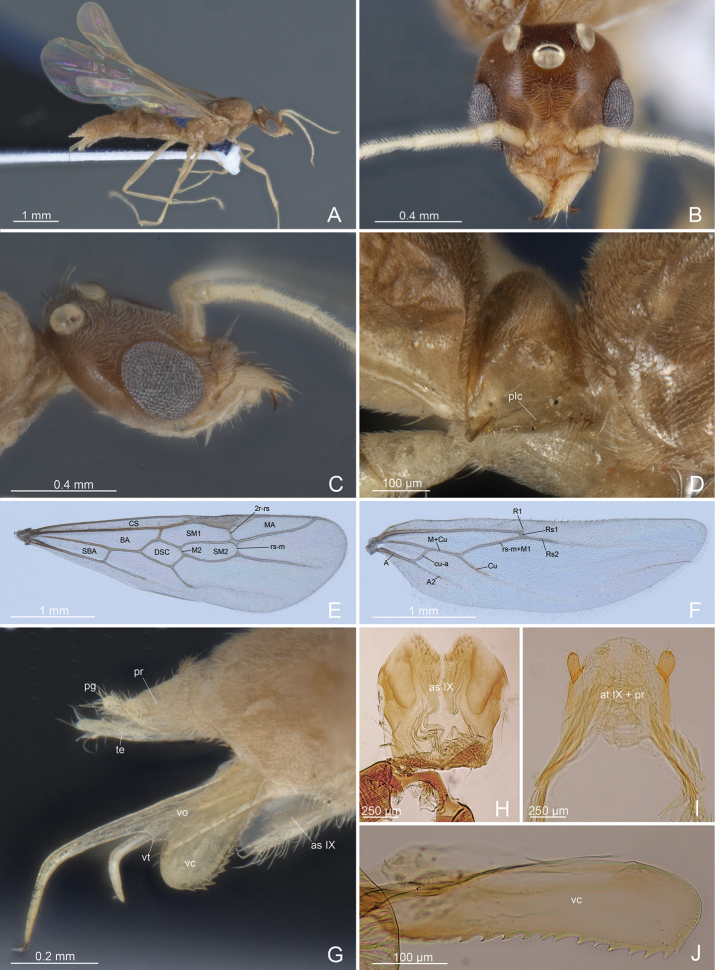
*L.paulistana* sp. nov.: **A** habitus **B** head in dorsal view **C** head in lateral view **D** petiole in lateral view **E** forewing **F** hindwing **G** external genitalia in lateral view **H** abdominal sternite IX **I** tergite IX+proctiger **J** valviceps lamina. Abbreviations: A: anal vein; atIX: abdominal tergite IX; asIX: abdominal sternite IX; BA: basal cell; CS: costal cell; Cu: cubital vein; cu-a: cubito-anal crossvein; DSC: discoidal cell; MA: marginal cell; M+Cu: medio-cubital vein; plc: petiole ventral postero-lateral carina; pg: pygostyle; pr: proctiger; Rs1: radial sector 1 vein; Rs2: radial sector 2 vein; rs-m+M1: radial sector-media crossvein; rs-m: radial sector-media cross-vein; 2r-rs: 2 radius-radial sector crossvein; R1: Radius 1; Rs1: Radial sector 1; Rs2: Radial sector 2; SBA: subbasal cell; SM1: submarginal 1 cell; SM2: submarginal cell 2; te: telomere; vc: valviceps; vo: volsella; vt: volsellar tooth.

**Figure 2.**
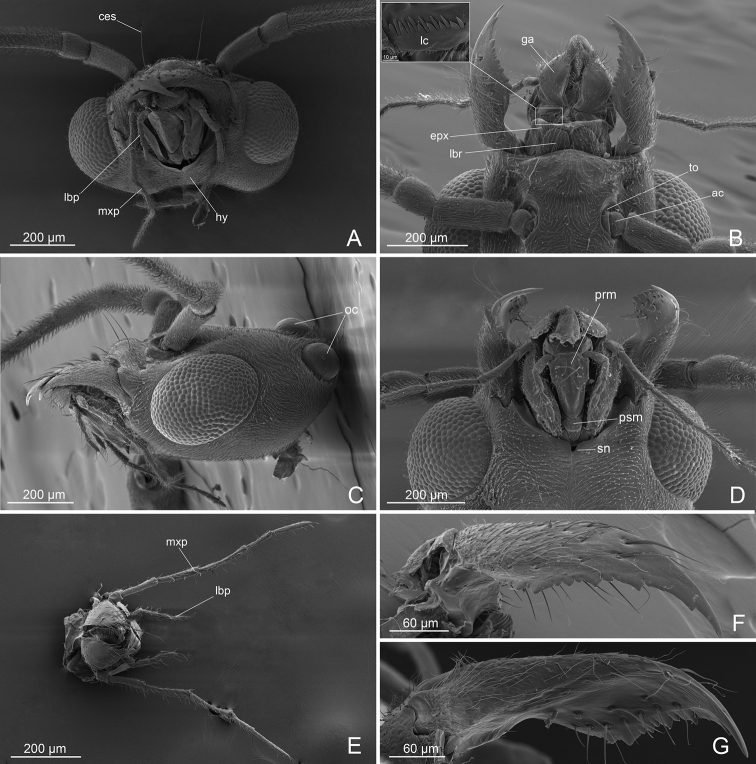
*L.paulistana* sp. nov.: **A** head oral view **B** head dorsal view **C** head lateral view **D** head ventral view **E** maxillar palp and labial palp **F**, **G** mandible. Abbreviations: ac: antennal condyle; ces: clypeal long erect setae; epx: epipharynx; ga: galea; hy: hypostoma; lbp: labial palp; lbr: labrum; lc: lacinia comb; mxp: maxilar palp; oc: ocelli; psm: postmentum; prm: prementum; sn: stipital notch; to: torulus.

**Figure 3.**
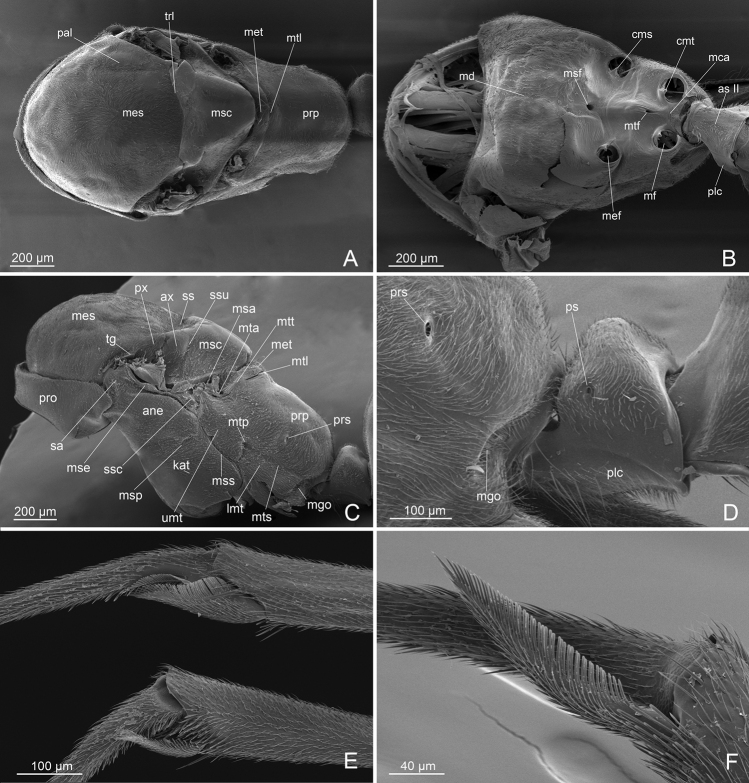
*L.paulistana* sp. nov.: **A** mesosoma dorsal view **B** mesosoma ventral view **C** mesosoma dorsal view **D** tegula **E** petiole **F** protibial spur and mesotibial spur **G** metatibial spur. Abbreviations: ane: anepisternum; asII: abdominal sternum II; ax: axilla; cms: medial coxal articular process of the mesopectus; cmt: medial coxal articular process of the metapectus; lmt: lower metapleuron; kat: katespisternum; mca: medial coxal articular process of the metapectus; md: mesodiscrimen; mef: mesocoxal foramen; mf: metacoxal foramen; mes: mesoscutum; msc: mesoscutellum; met: metascutellum; mgo: metapleural gland orifice; msa: mesoscutellar arm; mse: mesepimeron; msf: mesoprefurcal pit; mtf: metaprefurcal pit; msp: mesopleural pit; mss: mesopleural suture; mta: metascutellar arm; mtl: metascutellar line; mtp: metatentorial pit; mts: metapleuropropodeal suture; mtt: metascutellar trough; pal: parapsidal line; plc: petiole ventral posterolateral carina; pro: pronotum; prp: propodeum; prs: propodeal spiracle; ps: petiole spiracle; px: preaxilla; sa: subalar area; ss: scutoscutellar sulcus; ssc: spiracular sclerite; ssu: scutoscutellar suture; trl: transscutal line; tg: tegula; umt: upper metapleuron.

**Figure 4.**
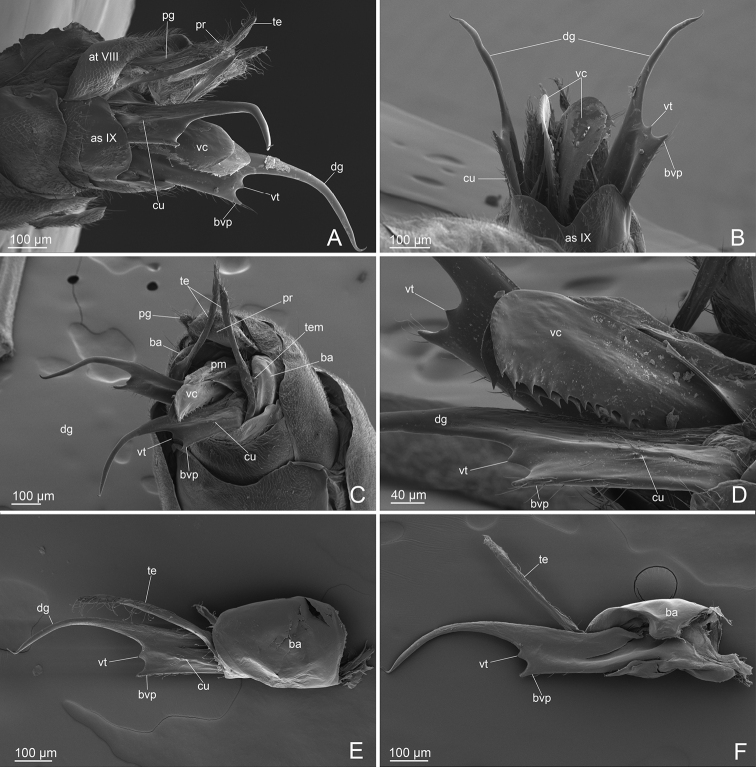
*L.paulistana* sp. nov.: **A** external genitalia lateral view **B** external genitalia ventral view **C** external genitalia ventro-lateral view **D** volsella and valviceps lateral view **E** basimere and volsella in lateral view **F** basimere and volsella in medial view. Abbreviations: asIX: abdominal sternite IX; atVIII: abdominal tergite VIII; pr: proctiger; ba: basimere; bvp: basivolsellar process; cu: cuspis; dg: digitus; pg: pygostyle; pm: penisvalve membrane; te: telomere; tem: telomere membrane; vc: valviceps; vo: volsella; vt: volsellar tooth.

**Figure 5.**
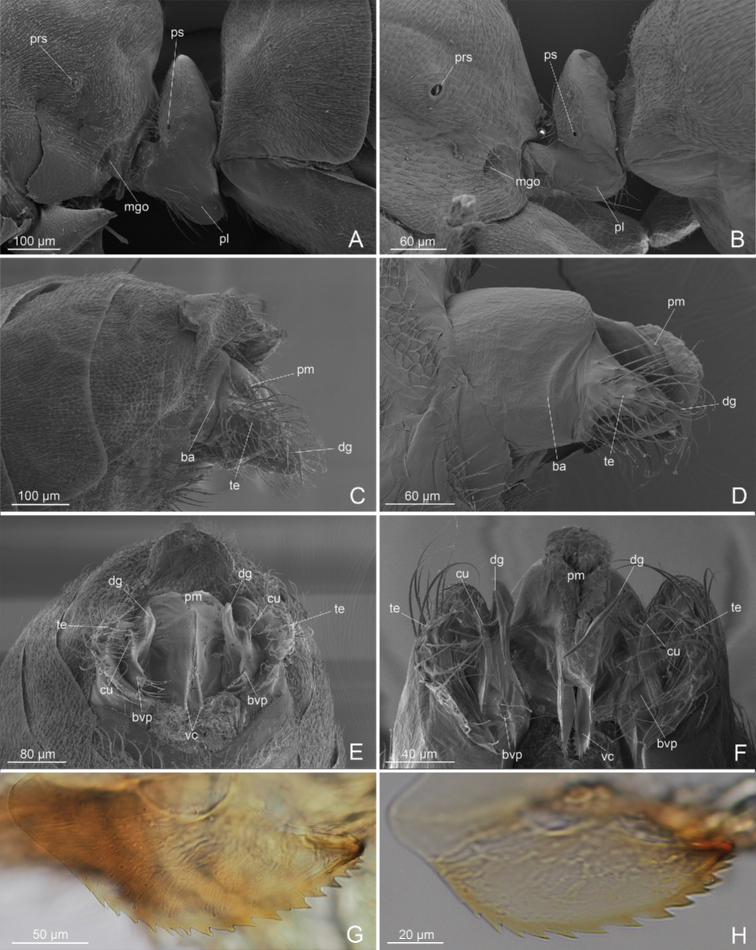
*L.humile*: **A** petiole in lateral view **C** external genitalia in lateral view **E** external genitalia in posterior view **G** valviceps lamina. *L.neotropicum***B** petiole in lateral view **D** external genitalia in lateral view **F** external genitalia in posterior view **H** valviceps lamina. Abbreviations: ba: basimere; bvp: basivolsellar process; cu: cuspis; dg: digitus; mgo: metapleural gland orifice; ps: petiole spiracle; prs: propodeal spiracle; pl: ventral petiolar process; pm: penisvalve membrane; te: telomere; vc: valviceps lamina.

**Figure 6.**
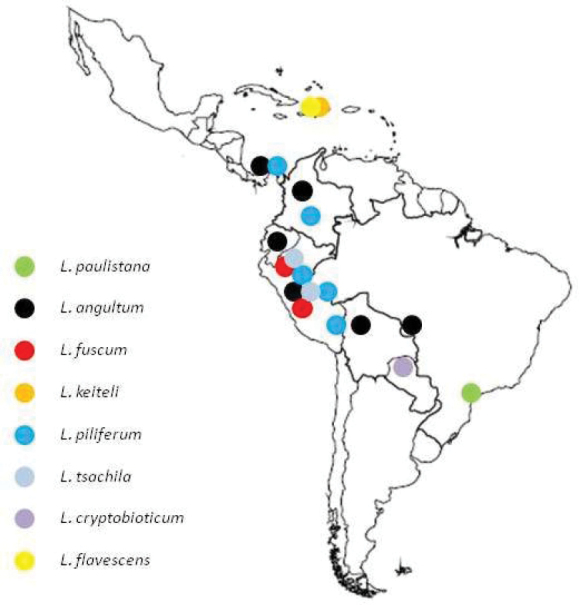
Distribution map of the *Linepithemafuscum* group of species in Central and South America.

Figs 1 , 2 , 3 , 4 , 5 , 6

####### Type material.

***Holotype*** male: Brazil, São Paulo city, 07–13 July 2013, light trap. Museum of Zoology of the Roma Tre University (Rome, Italy), MZUR3-HF0001.

***Paratypes***: Same data as holotype, 10 specimens deposited in the Museum of Zoology of the Roma Tre University (Rome, Italy), MZUR3-HF0002 to MZUR3-HF0008.

####### Holotype male description.

***Measurements* (in mm)**: HL: 0.70; HW: 0.65; MOD: 0.15; SL: 0.19; FL: 1.10; LHT: 1.11; LHF: 1.36; LHTa: 1.1; EL: 0.35; EW: 0.25; MML: 1.56; WL: 4.05; WHL: 3.28.

***Indices***: ES: 8.75; CI: 92.9; SI: 27.1; OI: 50.0; WI: 26.0; FI: 70.5.

***Male diagnosis***: notauli absent; forewing with two submarginal cells, marginal cell closed; petiole without ventral process; proctiger well developed; telomere narrow and elongated, digitiform distally; digitus dorsally long, spine-like distally; basivolsellar process reduced ventrally; volsellar tooth present distally between digitus and basivolsellar process; cuspis laterally very reduced.

***Habitus*** (Fig. 1A): slender ant, with metasoma elongated, longer than mesosoma. Dense pubescence present throughout the body, with sparse elongate, erected setae on head and mesosoma. Color of head and body medium brown; antennae and legs yellowish.

***Head*** (Figs 1B, C, 2A–G): longer that broad in full face view; with pubescence; eyes relatively large, occupying much of the anterolateral side of the head, separated from the insertion of the mandibles by a distance less than 1/3 than the length of the antennal scape. Two very long erect setae posterior to the median ocellus. Antennae filiform with 13 articles, regularly tapering to the apex; scape short, ~ 1/2 the length of the second funicular article; first article of the funiculus very short and cylindric, barrel shaped, ~ 1/3 the length of the second; second funicular article longest; articles of funiculus 9–12 short; antennal condylar bulb with series of spiniform Böhm sensilla, scape and first article of funiculus with recumbent sensilla; articles of funiculus 2–12 with erected sensilla. Anterior clypeal margin convex medially, with erect setae; medial part of the clypeus with two long erect setae. Labrum small, bilobed, separated by a deep notch, each lobe laterally reduced and pointed and medially produced into a round plate dorsally with multiple setae. Epipharynx unsclerotized, exceeding the length of labrum and distinctly visible in dorsal view. Mandibles large, with masticatory margin long and broad; apical tooth long and pointed; preapical tooth short and subtriangular, followed by a series of teeth and denticles; basal margin short and strongly diverging; basal part of the mandible covered by dense pubescence on dorsal and lateral surface; masticatory margin without pubescence, with very long setae (6 or 7 dorsally and 9–11 ventrally). Maxillae with subrectangular stripes, pubescent dorsolaterally and not setose ventrally. Maxillary palps with six articles, together with a total length that exceeds half the length of head; article I very short, II subequal in length and diameter to III; IV subequal in length and minor in diameter to III; V and VI thin and short. Dorsal surface of galea not pubescent, with short, scattered setae basally, long setae laterally and distally; ental margin of galea with maxillary comb medially projected. Lacinia comb composed of teeth alternating with stout setae. Labium with prementum subrectangular and elongate, wider and with two very long setae ventrally; postmentum with spinulate microsculpture; glossa with comb-like, backward directed fringes; labial palps of four articles: article I subequal in length to II; III and IV shorter and thinner than II. Hypostoma with anteromedial stipital notch.

***Mesosoma*** (Figs 1A, D–F, 3A–D, F, G): shorter in length than metasoma. Pronotum short, with recumbent pubescence, laterally projecting over the anepisternum. Mesoscutum strongly convex in lateral view, not overhanging the pronotum, totally covered by recumbent pubescence; notauli absent; parapsidal lines evident. Mesoscutellum swelling, separated from mesoscutum by a deep scutoscutellar sulcus, smooth dorsally and with recumbent pubescence laterally. Metascutellum convex, lower than mesoscutellum and not overlapping with the propodeum, with recumbent pubescence and long setae dorsomedially. Metapleural gland orifice very large and posterolateral. Forewings (WL: 4,05; WI: 25,95) with two submarginal cells, discoidal cell, marginal cell closed and large, dark pterostigma, two radius-radial sector cross-vein almost in line with radial sector-media, two media vein present; tegula with row of small hairs. Hindwings (WHL: 3,27) without two medial vein; one radius vein nebulous and one radial sector present but short; 8–11 hamuli. Legs (FI: 70,5); profemur (FL: 1,10); metafemur (LHF: 1,36); metatibia (LHT: 1,11) with single apical very long spur pectinate on the inner margin and with small cuticular fringes on the external margin; metabasitarsus long (LHTa: 1,09); mesotibia with short apical spur pectinate; pretarsal claw simple with arolium and planta developed. Propodeum in lateral view slightly convex dorsally and straight posteriorly; propodeal spiracle orifice lateral and rounded. Sternal region as in Fig. 3B.

***Metasoma*** (Figs 1A, D, 3D): Petiole erect, taller than wide in lateral view and rounded dorsally, anterior profile convex and posterior concave; setae and pubescence present in the anterior face, posterior face without setae or pubescence; petiolar tergosternal suture with small posterior lobe; ventral profile of petiole only slightly convex, without subpetiolar process, with long setae anteriorly and erect setae posteriorly; petiole articulated ventrally with abdominal segment III. Gaster elongate, with dense pubescence on tergites and sternites, hairs of pubescence longer on last sternites where long setae are also present laterally; pygostyles very long and apical erected setae; proctiger well developed, extending posteriorly beyond the IX abdominal tergite in the form of a large lamina, straight distally and light colored.

***External genitalia*** (Figs 1G–J, 4A–F): IX abdominal sternite bilobed distally, due to a deep medial notch. Paramere composed of a short basimere, dish-like, strongly thinned dorsally, and shortened distally, and telomere narrow and very long ribbon-like, digitiform distally, that extends postero-dorsally overcoming the proctiger. Volsella composed of i) parossiculus (basivolsella+cuspis) with small and pointed ventral basivolsellar process and with apical and ventral setae along the edge; lateral cuspis very reduced to a ridge, bearing long setae on apical part; ii) very long falcate dorsal digitus, downturned, distally spine-like; iii) triangular volsellar tooth posteromedially placed, between basivolsellar process and digitus, without setae. Penis valve very long with valviceps lamina ventrally straight and multidentate with 13 or 14 teeth, apically rounded.

####### Remarks.

Queen and worker unknown. Currently only known from São Paulo city, Brazil. Mating flight January to August.

####### Derivatio nominis.

The name *paulistana* refers to the Brazilian appellation of the citizens of São Paulo city, where several males of the new species were captured.

### ﻿Description of the male external genitalia of *Linepithemahumile* (*humile* group) and *L.neotropicum* (*neotropicum* group)

External genitalia (Fig. 5A–F). The two species *L.humile* and *L.neotropicum* show a similar structure of the following features: IX abdominal sternite distally concave medially; paramere composed of a short dish-like basimere, which extends dorsally, and a distally lobate telomere; volsella composed of: i) parossiculus (basivolsella+cuspis) with posteroventral basivolsellar process developed, lobate, with long setae along its margin and lateral cuspis developed, with apical setae; ii) median falcate digitus, downturned, distally spine-like and slightly longer than the telomere; penisvalve with valviceps lamina ventrally strongly convex, rounded and multidentate with 16 or 17 teeth in *L.humile* and 13 teeth in *L.neotropicum*. The two species are clearly distinguished by the shape of the valviceps lamina and for structure of the volsella, that in *L.neotropicum* is characterized by having a lateral cuspis lobate apically and parallel to the median digitus; instead in *L.humile* the lateral cuspis is slightly pointed apically and more ventral than the digitus.

In Table 1 we report an updated and comparative list of the most relevant diagnostic characters that differentiate the males of the *Linepithemafuscum* group from the males of the other species of the same genus.

#### ﻿Morphometrics

See Table 2.

**Table 2.** Most relevant morphometric male characters of *L.paulistana* sp. nov. and species of the *Linepithemafuscum*-group. The measurements of *L.angulatum*, *L.fuscum*, *L.keiteli*, *L.piliferum*, and *L.tsachila* are taken from Wild (2007). Abbreviations are listed in Materials and methods and follow the system of Wild (2007). *n* = number of specimens measured.

	*L.paulistana* sp. nov. (*n* = 10)	*L.angulatum* (*n* = 4)	*L.fuscum* (*n* = 5)	*L.keiteli* (*n* = 4)	*L.piliferum* (*n* = 5)	*L.tsachila* (*n* = 4)
HL	0.67–0.71	0.73–0.77	0.68–0.74	0.61–0.70	0.66–0.71	0.71–0.74
HW	0.64–0.67	0.67–0.71	0.63–0.70	0.56–0.65	0.63–0.78	0.69–0.71
SL	0.19–0.20	0.20–0.21	0.19–0.21	0.21–0.22	0.23–0.24	0.21–0.24
FL	1.07–1.11	1.13–1.25	1.01–1.07	0.88–1.06	0.91–1.03	1.10–1.15
LHT	1.06–1.12	1.09–1.25	1.01–1.07	0.85–1.10	0.90–1.02	1.09–1.13
EL	0.33–0.37	0.32–0.35	0.32–0.37	0.24–0.28	0.34–0.37	0.39–0.43
MML	1.46–1.58	0.76–0.95	1.48–1.67	1.31–1.61	1.44–1.57	1.53–1.62
WL	4.03–4.14	4.1–4.75	4.16–4.49	3.66–4.68	4.4–4.9	4.35–4.51
CI	88–93	92–95	92–97	85–98	90–97	93–97
SI	26–29	27–30	27–30	31–34	32–35	30–32
OI	47–52	43–47	48–52	37–42	51–53	55–58
WI	25–27	26–28	26–28	28–29	29–31	27–28
FI	66–69	70–73	63–68	66–68	61–66	70–71

#### ﻿Geographic distribution

The species of the *fuscum* group are geographically distributed as follows: *L.angulatum* Emery, 1894, in Costa Rica south, west South America to the Brazilian Pantanal; *L.fuscum* Mayr, 1866, in Peru and Ecuador; *L.keiteli* Forel, 1906, in Hispaniola; *L.piliferum* Mayr, 1870, in mountains of northwestern South America to Costa Rica; *L.tsachila* Wild, 2007, in Colombia and Ecuador; *L.cryptobioticum* Wild, 2007, Paraguai; *L.flavescens* Wheeler & Mann, 1914, Hispaniola (Wild 2007; Escarga and Guerrero 2016); and *L.paulistana* sp. nov. São Paulo, Brazil (Fig. 6).

### ﻿Key

**Figure 7.**
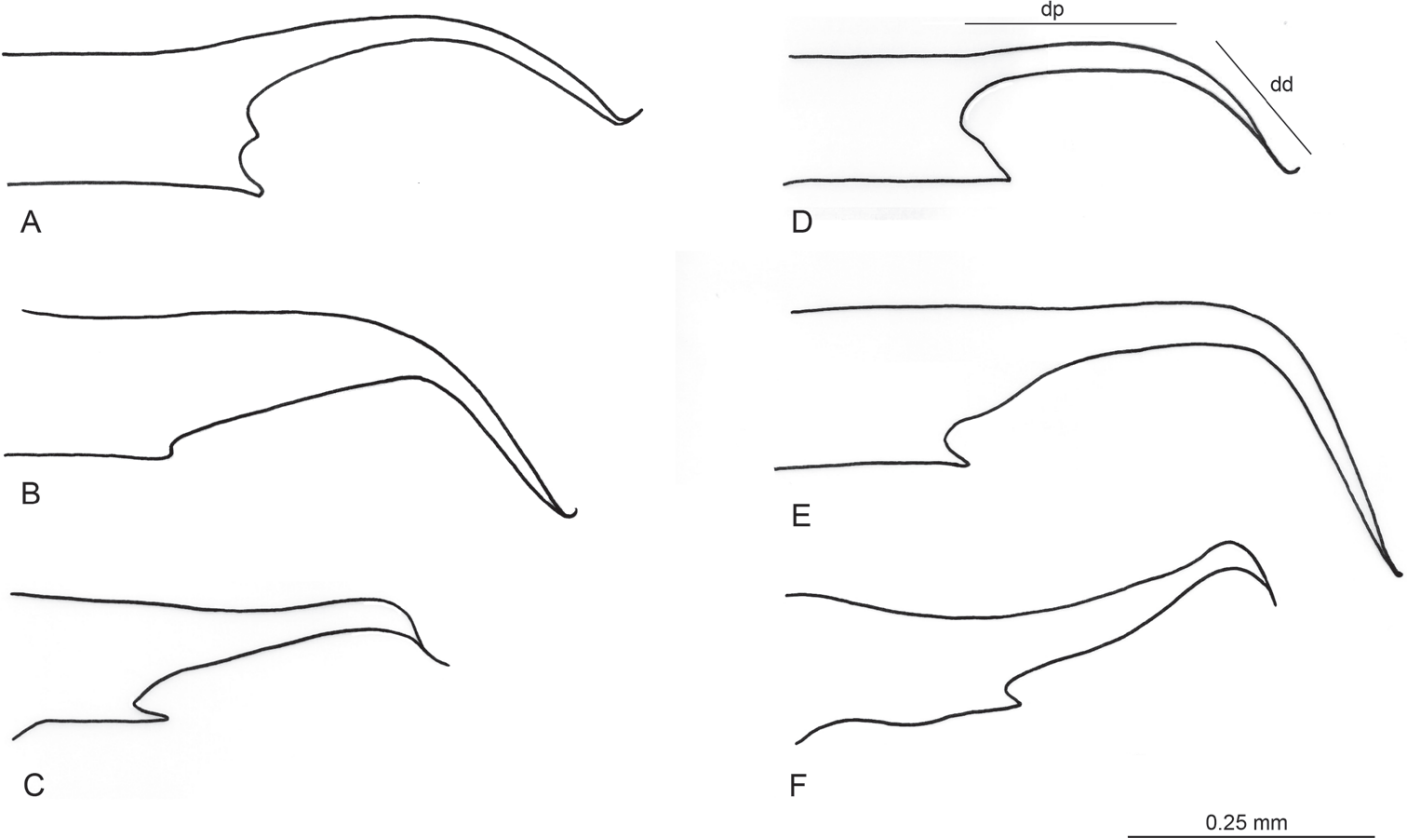
Shape of volsella in males of the *Linepithema* Fuscum-group **A***L.paulistana* sp. nov. **B***L.angulatum***C***L.piliferum***D***L.fuscum***E***L.keiteli***F***L.tsachila*. Abbreviations: dp = proximal arm of digitus; dd = distal arm of digitus. **B–F** re-drawn from Wild (2007).

A dichotomous key to identify the males of known species of the *fuscum* group is presented. The form of the volsella in the species of *Linepithemafuscum* group is the main morphological feature used by Wild (2007) to differentiate the species. The digitus was divided by Wild (2007) into proximal arm and distal arm (Fig. 7D). We follow this criterion, which gives the possibility to compare this new species with the other described species of the *fuscum* group (Fig. 7). In *L.paulistana* sp. nov. the distal arm is similar in length to the proximal arm.

**Table:** 

1	Forewings with one submarginal cell; petiole with ventral process well developed (Fig. 5A, B); pygostyle short (Fig. 5C); proctiger slightly developed (Fig. 5E, F); telomere short and lobiform (Fig. 5C, D); lateral cuspis developed (Fig. 5E, F); ventral basivolsellar process developed (Fig. 5E, F)	***Linepithema* species not in the *fuscum* group**
–	Forewings with two submarginal cells (Fig. 1E); petiole with ventral process slightly developed consisting of a slight convexity (Figs 1D, 3D); pygostyle very long (Fig. 4A); proctiger well developed (Fig. 1G, I); telomere very long and narrow (Fig. 4C); lateral cuspis very reduced (Fig. 4E); ventral basivolsellar process reduced (Fig. 4D)	***Linepithemafuscum* group 2**
2	Digitus very long, downturned, falcate (Fig. 7A, B, D, E)	**3**
–	Digitus moderately long, not downturned, not falcate (Fig. 7C, F)	**6**
3	Basivolsellar process not pointed apically (Fig. 7B)	***L.angulatum***
–	Basivolsellar process pointed apically (Fig. 7A, D, E)	**4**
4	Digitus downturned with almost a 90° angle (Fig. 7E); ocular index OI = 37–42; Hispaniola island	***L.keiteli***
–	Digitus downturned with 45° angle (Fig. 7A, D); ocular index OI = 47–52	**5**
5	Volsellar tooth present distally between basivolsellar process and digitus (Fig. 7A); São Paulo, Brazil	***L.paulistana* sp. nov.**
–	Volsellar tooth absent (Fig. 7D); Peru and Ecuador	***L.fuscum***
6	Digitus strongly concave dorsally (Fig. 7F)	***L.tsachila***
–	Digitus straight to slightly concave (Fig. 7C)	***L.piliferum***

## ﻿Discussion

The genus *Linepithema* was designated by Mayr (1866) for males that he described as *L.fuscum*, for which workers and queens remain unknown. Regardless, males of the *fuscum* group are easily distinguished from other *Linepithema* species due to the synapomorphic features indicated by Shattuck (1992b) and Wild (2007). We report these features in Table 1 together with new or updated characters, while in Table 2 we report the male morphometrics for all species of the *L.fuscum* group with the addition of our proposed new species. Currently, males of the *L.fuscum* group are known for five out of seven species and are recognizable by the peculiar morphology of the volsella (digitus and basivolsellar process, Fig. 6), which was used as the main species-specific feature by Wild (2007) in his dichotomous key to males. *Linepithemapaulistana* sp. nov. shows all diagnostic features of *fuscum* group and is distinguished from the other species by the presence of a triangular tooth situated on the volsella distally, between digitus and basivolsellar process (Figs 1G, 4A–F). For this reason, in the present description of *L.paulistana* sp. nov. we emphasized the morphology of the external genitalia (in particular of the volsella).

The first description of the external genitalia in *L.fuscum* was made by Mayr (1866), later revised by Emery (1912), who supplied a new description of all the three paired valves, figured by a schematic drawing. Subsequently, the external genitalia of the genus *Linepithemafuscum* group have been described by Shattuck (1992b) and Wild (2007). According to careful studies of several authors, the volsella is composed of two separate sclerites: the lateroventral parossiculus and the medial digitus (Snodgrass 1941); both sclerites are united longitudinally by a narrow, sclerotized suture (Schulmeister 2001); the parossiculus is a unique sclerite (Schulmeister 2001), consisting in a basal basivolsella, a ventral basivolsellar process and a lateral cuspis (Snodgrass 1941). The distal apex of the cuspis and the ventral and apical basivolsellar process are usually recognizable by the presence of setae (Boudinot 2013). By using SEM and optical microscopy we analyzed the external genitalia of *L.paulistana* sp. nov. and re-evaluated some characters and previous interpretations in the *Linepithemafuscum* group.

In *L.paulistana* sp. nov. we found that: i) the cuspis is very reduced but clearly recognizable as a ridge by the presence of long setae on apical part (Fig. 4C–E), probably due to a lateromedial flattening of the volsella, and not absent as claimed by Shattuck (1992b) and Wild (2007) in the diagnosis of the *fuscum* group. In our opinion, the presence of the cuspis in males of other species of the *fuscum* group should be re-considered. ii) The “ventrodistal process” of the volsella described by Wild (2007) is the basivolsellar process, which is reduced and with apical and ventral setae (Fig. 4A–F). The *L.fuscum* group is, to our knowledge, an exception in the subfamily Dolichoderinae: the paramere shows a telomere very elongated, narrow, ribbon-like and digitiform apically, extending anterodorsally up the proctiger (Fig. 4A–C). Otherwise, the external genitalia of the subfamily Dolichoderinae, based on the diagnosis by Boudinot (2015), is characterized by a paramere, with basimere dorsally and distally developed, and a telomere strongly reduced and lobed, representing a synapomorphy of the subfamily (Yoshimura and Fisher 2011; Boudinot 2015). As for the paramere (basimere+telomere), in the males of the *fuscum* group, including *L.paulistana* sp. nov., the term telomere should be preferred to gonostylus, utilized in the previous descriptions (Shattuck 1992b), consistently with the same lobate structures of other dolichoderine males, as suggested by Boudinot (2013).

In order to show the morphological diversity of the male external genitalia within the *Linepithema* species-groups, we also analyzed and illustrated by SEM the species *L.humile* (representative of *humile* group) and *L.neotropicum* (representative of the *neotropicum* group), and we compared these species with *L.paulistana* sp. nov. (representative of the *fuscum* group). We highlighted, in particular, that: i) in *L.humile* and *L.neotropicum* the basivolsellar process is well developed, located ventrally, with lateral cuspis and medial digitus (Fig. 5C–F). In males of the *fuscum* group, due to elongation and flattening lateromedially of the volsella, the cuspis is reduced laterally to the digitus and the basivolsellar process is reduced ventrally (Fig. 4A–F). ii) The morphology of the dentate valviceps lamina in *L.humile* and *L.neotropicum* has a rounded profile dentate ventral edge strongly convex and rounded, while in *L.paulistana* sp. nov. has a dentate ventral edge straight (Figs 1J, 4D, 5G, H). iii) The proctiger in *L.paulistana* sp. nov. is well developed (Figs 1G, I, 4A, C), probably representing a morphological character of the *fuscum* group, given that in *L.tsachila* and *L.fuscum* is present (AntWeb 2022). The proctiger is very reduced in the males of the other *Linepithema* species-groups (Fig. 5C, D). Relevant morphological characters that distinguish the species of the *Linepithemafuscum* group from the other species of the same genus are reported in Table 1.

Concerning the morphometric characters reported in Table 2, *L.paulistana* sp. nov. shows several similarities with *L.fuscum*, but differs in having smaller head (CI), much smaller wings (WL), and longer profemora (FL). However, the possibility that *L.paulistana* sp. nov. could represent an allopatric population of *L.fuscum*, with some morphological differences, seems highly unlikely due to: i) different morphology and dimensions of the volsella, an acknowledged species-specific character; ii) the great geographic distance between the species, *L.fuscum* from the Pacific side and *L.paulistana* sp. nov. from of the Atlantic side, separated by the Andes Mountains; iii) the different climatic and ecological conditions where the two species live.

As the *fuscum* group is male-based, the assignment of the species to this group should be verified by using male characters. However, based on worker morphology (Wild 2007), two species of *Linepithema* with unknown males, *L.flavescens* and *L.cryptobioticum*, were assigned to the *fuscum* group (Wild 2007). For *L.cryptobioticum* this assignment was supported by a molecular study (Wild 2009). Since the workers of the new species here described are still unknown, we cannot exclude *a priori* the possibility that males of *L.paulistana* sp. nov. could be part of the aforementioned species. However, this is unlikely based on the following arguments: 1) The species *L.cryptobioticum*, *L.flavescens* and *L.paulistana* sp. nov. have allopatric distribution: *L.flavescens* is very far from the other species and shows very localized distribution in the Northern hemisphere, being only present in the Hispaniola Island; *L.cryptobioticum* is only present in Paraguay; *L.paulistana* sp. nov. is only present in São Paulo city. 2) The species *L.cryptobioticum* was included in the molecular phylogeny of Wild (2009) and was found to be a daughter species of *L.angulatum*. For this reason, we hypothesize that male of *L.cryptobioticum* is morphologically very similar to that of *L.angulatum*. Comparing the males of *L.angulatum* and *L.paulistana* sp. nov. we find the following distinctive differences that widely reduce the possibility of a close relationship between the two species: the volsella is very different in shape (Fig. 7A, B) and the morphometric dimensions are very different (see for example HL, HW, FL, MML, OI in Table 2). However, we cannot exclude that *L.paulistana* sp. nov. is a derived offshoot within the “*angulatum*” cluster. For these reasons, in order to have more clarity about the relationships in this intricate species group, we will have to wait until the discovery of males of *L.cryptobioticum*, as well as *L.angulatum* males from other representative localities within the wide range of distribution, spanning from Meso-America to the Pantanal.

Finally, it is remarkable that *L.paulistana* sp. nov. is the only species of the *fuscum* group present in the eastern part of South America, extending the former distribution of this species group (Fig. 6), widely distributed i western South America. This finding represents an important range expansion of the genus, which opens the possibility to find additional species of *L.fuscum* group also in other parts of eastern South America.

## ﻿Conclusions

The present work confirms that the information on morphological characters of male, especially of male external genitalia, is effective for the identification of ant genera and species, as in most insects (Bolton 2003; Yoshimura and Fisher 2007; Yoshimura and Fisher 2011; Boudinot 2013, 2015; Cantone 2017). We are strongly convinced that a greater knowledge of the male caste would greatly help the identification of most ant species, genera, or species groups, like *Linepithema*, where workers are very difficult to identify at the species level. Unfortunately, in the family Formicidae the knowledge on the male caste is very limited and the taxonomy is traditionally mainly based on workers. *Linepithema* is one of the very rare genera described on male caste and allows to describe a new species, like *L.paulistana* sp. nov., based on male specimens. In our opinion the great morphological differences between the external genitalia of species of the *fuscum* group and all the other species formerly assigned to the genus *Iridomyrmex*, like *L.humile*, and now included in three different *Linepithema* species groups, suggest that a re-evaluation of the taxonomic status of this genus is urgently needed.

## Supplementary Material

XML Treatment for Linepithema paulistana
